# Haemoadsorption to remove inflammatory mediators in sepsis: past, present, and future

**DOI:** 10.1186/s40635-025-00740-0

**Published:** 2025-03-21

**Authors:** Nicole J. B. Waalders, Matthijs Kox, Peter Pickkers

**Affiliations:** 1 Department of Intensive Care Medicine, Radboud university medical center, Nijmegen, The Netherlands; 2 Radboud university medical center, Radboud Center for Infectious Diseases (RCI), Nijmegen, The Netherlands

**Keywords:** Sepsis, Septic shock, Hemoadsorption, Hemofiltration, Blood purification, Cytokines

## Abstract

While a dysregulated immune response is at the center of the sepsis definition, standard care is still solely focussed on prompt administration of antimicrobial therapy, source control, resuscitation and organ supportive therapies. Extracorporeal blood purification therapies, such as haemoadsorption, have been proposed as a possible adjunctive therapy to standard care in sepsis. These adsorption devices aim to rebalance the dysregulated immune response by removal of excessive amounts of circulating inflammatory mediators, including cytokines and endotoxins. Thus far, the effects of haemoadsorption on clinical outcomes have been insufficiently studied and although its routine use is not justified based on the current evidence, multiple centers use these devices in patients with severe septic shock. This narrative review describes the most well-studied adsorption devices as well as a novel selective adsorption device called the ‘IL-6-Sieve’, including in vitro data showing its capturing potential. Finally, it addresses important considerations for future trials on haemoadsorption in septic patients.

## Take-home message

Haemoadsorption may improve outcomes of patients with sepsis by attenuating the inflammatory response, but current evidence does not support their routine use, therefore selective adsorption therapy that may enable removal of specific mediators represents a novel approach. Future studies in this area should incorporate predictive enrichment strategies to select those patients that may benefit the most, focus on optimal timing and application of therapy.

## Introduction

Sepsis remains one of the leading causes of deaths worldwide. The most recent data indicates that approximately 20% of all deaths are associated with sepsis [1]. Sepsis is a heterogeneous syndrome with a complex pathophysiology. The dysregulated immune response in patients with sepsis ranges from an overzealous hyperinflammatory state with excessive cytokine production to a profound immunosuppressed phenotype also known as sepsis-induced immunoparalysis [2, 3]. Over the years, several pharmacological interventions aimed at modulating the immune response have been investigated, with predominantly disappointing results [4]. Extracorporeal blood purification (EBP) therapies have also been proposed as a possible adjunctive therapy to rebalance the immune response by removal of inflammatory mediators, such as cytokines, and/or pathogen-associated molecular patterns such as endotoxins [5, 6]. EBP consist of either filtration [e.g. high-volume haemofiltration (HVHF)], diffusion (e.g. continuous venovenous haemodialysis), adsorption (i.e. haemoadsorption; previously referred to as haemoperfusion), or a combination of these (e.g. continuous plasma filtration coupled with adsorption) [7–9].

## Mechanisms underlying the proposed beneficial effects of extracorporeal blood purification

A number of theories explaining the putative beneficial effects of EBP have been proposed over the last decades. First, the ‘peak cytokine hypothesis’ was described [10, 11], in which EBP attenuates the relative excess of pro- and anti-inflammatory mediators in the circulation. In turn, this could directly mitigate organ damage and mortality. Another theory is the ‘threshold immunomodulation hypothesis’ [12]: removal of circulating cytokines by EBP may drain the inflammatory milieu in tissues, based on an equilibrium of cytokine concentrations between these two compartments. The subsequent drainage of cytokines from tissues could alter the local immune cascade in a favourable way and prevent further organ damage. Along the same lines, it was suggested that by removing plasma cytokines and thereby increasing the blood-tissue gradient, EBP may enhance migration of neutrophils to the infected tissue, and improve antigen-presenting capabilities [13], as well as leukocyte recruitment, oxidative burst and phagocytosis, and leukocyte responsiveness [14, 15]. This theory resembles the ‘cytokinetic model’, in which the abovementioned cytokine concentration gradient between the circulation and the infected tissue promotes leukocyte chemotaxis and bacterial clearance in the tissue [5, 16]. Specific for HVHF, the ‘mediator delivery hypothesis’ was postulated [17], in which high volumes of crystalloids fluids are used to replace fluid loss and induce lymphatic flow. The high volume of ‘replacement fluids’ may drive mediators from the tissue interstitium to the lymphatic system, after which they are transported to the circulation and removed by a dialysis filter or by metabolic clearance.

In the present work, we focus on haemoadsorption, as this represents the most direct method to remove inflammatory mediators.

## Currently available adsorption-based therapies

**Fig. 1:**
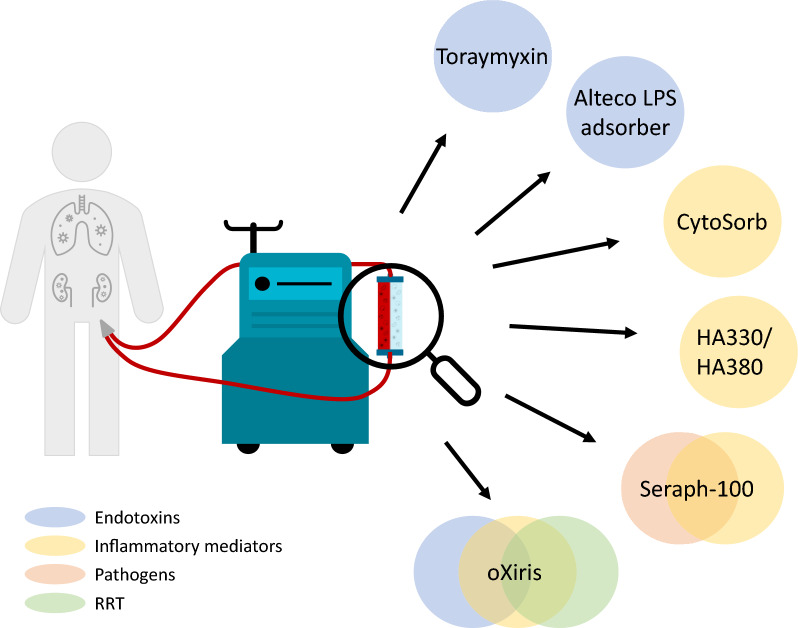
Schematic overview of different adsorption-based therapies in critically ill septic patients. *LPS* lipopolysaccharide, *RRT* renal replacement therapy

In the past, adsorption devices consisted of materials such as charcoal and were mainly used in case of acute poisoning or (drug) intoxication [18, 19]. More recently, several biocompatible adsorption devices aimed at removing circulating inflammatory mediators have been developed. These devices consist of adsorbing materials that bind mediators through van der Waals, electrostatic and/or hydrophobic interactions [20]. Adsorption to the device is also influenced by differences in the structure and diameter of the pores inside the adsorber, as well as the blood flow rate [20–22]. In this paragraph, we present the characteristics and (clinical) effects of the most well-studied adsorption devices, ranked according to the amount of available evidence. A schematic overview of these therapies is depicted in Fig. 1 and the available evidence obtained in patient studies that is included in this review is summarised in Table 1.

**Table 1:** Overview of the current evidence of adsorption devices obtained in in patient studies

Authors	Study design	Control group	Total number of patients	Disease or surgery	Primary outcome	Secondary outcomes	Comments
Toraymyxin							
Cruz et al. [26]	RCT	Standard care	64	Severe sepsis or septic shock of abdominal origin	Increase in MAP, decrease in inotropic score	Improved SOFA score and reduced 28-day mortality in a Cox proportional hazard model	No difference in crude 28-day mortality [27]
Cutuli et al. [28]	Retrospective; registry	None	357	Gram-negative related severe sepsis or septic shock	Improved cardiovascular, respiratory and renal components of the SOFA score		–
Nakamura et al. [29]	Retrospective; propensity score matched	Standard care	1723	Septic shock	Lower all-cause mortality	More ICU-free days. No difference in ICU mortality	–
Payen et al. [30]	RCT	Standard care	232	Septic shock and emergency abdominal surgery	No difference in 28-day mortality	No difference in in SOFA score and catecholamine infusion rate. No difference in mortality at days 3, 7 and 90	–
Dellinger et al. [31]	RCT	Sham filter	450	Septic shock and EAA ≥ 0.6	No difference in 28-day mortality in all patients or in patients with a high multiple organ dysfunction score	No difference in hospital stay, RRT-free days, mechanical ventilation-free days, serum creatinine, and multiple organ dysfunction score	Post-hoc analysis [38] showed potential benefit in patients with EAA 0.6–0.89
Srisawat et al. [32]	RCT	Standard care	59	Severe sepsis or septic shock and EAA ≥ 0.6	Increased mHLA-DR expression	No difference in inotropic score, vasopressor dose, EAA levels, 7- and 28-day mortality, and ICU and ventilator free days	–
CytoSorb							
Schädler et al. [51]	RCT	Standard care	97	Severe sepsis or septic shock	Elimination of IL-6 across the filter but no difference in circulating IL-6 concentrations	No difference in circulating concentrations of other cytokines, duration of mechanical ventilation, and multiple organ dysfunction score	–
Scharf et al. [52]	Retrospective; propensity score matched	Standard care	143	Critically ill patients with circulating IL-6 > 10,000 pg/mL	No difference in reduction of circulating IL-6 concentrations, norepinephrine requirement, and in-hospital mortality		–
Bernardi et al. [53]	RCT	Standard care	37	CPB surgery	No difference in circulating IL-6 concentrations. Higher circulating IL-10 concentrations	No difference in vasopressor requirement, CRP or PCT, and 30-day mortality	–
Hawchar et al. [55]	RCT	Standard care	20	Septic shock	No difference in SOFA scores and CRP. Lower PCT levels. Reduced norepinephrine requirement		
Jafron (HA330/HA380)							
Huang et al. [66]	RCT	Standard care	44	Severe sepsis	Attenuated circulating IL-6 and IL-8 concentrations. Improved hemodynamic parameters, lower SOFA score, shorter ICU LOS, and lower ICU mortality		–
Huang et al. [67]	RCT	Standard care	46	ARDS caused by extrapulmonary sepsis	Lower circulating IL-1 and TNF concentrations. Lower dopamine and norepinephrine requirements, shorter ICU LOS, lower ICU and 28-day mortality		–
He et al. [69]	RCT	Standard care	60	CPB surgery	Lower circulating TNF, IL-6, IL-8, and IL-10 concentrations and vasoactive inotropic score. Shorter ICU LOS and duration of mechanical ventilation		–
Wang et al. [70]	Retrospective	Standard care	117	Acute type A aortic dissection surgery	Lower circulating IL-6 concentrations and lower incidence of AKI and ARDS. No difference in postoperative complications and mortality		Effects on AKI and ARDS incidence should be interpreted with caution due to a low fragility index
oXiris							
Schwindenhammer et al. [78]	Retrospective	None	31	Septic shock requiring RRT	Improvement of lactate and reduced norepinephrine requirement. Hospital mortality lower than expected for the most severe patients		Patients with abdominal septic shock or those affected by Gram-negative bacteria appeared to benefit most
Broman et al. [79]	RCT; crossover	Standard care	16	Septic shock and AKI	More pronounced decrease in circulating endotoxin and cytokine concentrations. Lower lactate levels and reduced norepinephrine requirement		Results should be interpreted with caution due to baseline differences and regression to the mean
Wendel-Garcia et al. [80]	RCT	Toraymyxin or standard care	30	Severe septic shock and EAA ≥ 0.6	No difference in reduction of EAA levels	No difference in other inflammatory markers, SOFA score and lactate levels	–
Pérez-Fernández et al. [84]	RCT	Standard care	343	CPB surgery	Lower incidence of AKI	No difference in ICU LOS, hospital LOS, and mortality at day 7, 28 and 90	–
Alteco LPS adsorber							
Ala-Kokko et al. [87]	Observational case series	Standard care (historic)	24	Septic shock	Shorter duration of norepinephrine infusion, decrease in EAA activity		–
Shum et al. [88]	RCT	Standard care	15	Severe sepsis or septic shock of abdominal origin	No difference in SOFA scores	No difference in vasopressor requirements, ICU and hospital LOS, and ICU and 28-day mortality	Terminated prematurely due to lack of efficacy
Lipcsey et al. [89]	RCT	Sham filter	15	Septic shock	Safe. No difference in circulating endotoxin levels, concentrations of inflammatory mediators, organ dysfunction, mortality, and ICU LOS		Terminated prematurely due to low enrolment rate
Seraph-100							
Eden et al. [93]	Prospective; first-in-human trial	None	15	Chronic haemodialysis patients on RRT with infection	Safe	Time to positivity increased between inflow and outflow cultures	–
Chitty et al. [91]	Retrospective	Standard care	106	Severe COVID-19	More vasopressor-free days (not significant in a multivariable model)	Lower in-hospital mortality compared to internal control group (but not compared to an external control group)	–
Olson et al. [94]	Case series	None	2	Severe COVID-19	Decrease in vasopressor dose, temperature, circulating IL-6 and CRP		–
Pape et al. [95]	Case report	None	1	Severe COVID-19	Well tolerated. Decrease in inotropic support		–
Rifkin et al. [96]	Case series	None	4	Severe COVID-19	Safe and well tolerated. Decrease in CRP and PCT. Increase in PaO_2_/FiO_2_ ratio		–
Schmidt et al. [97]	Retrospective; registry	None	78	COVID-19	Well tolerated		–
CPFA							
Livigni et al. [99]	RCT	Standard care	184	Septic shock	No difference in hospital mortality	No difference in new-onset organ failure or ICU free days during the first 30 days	Terminated prematurely due to futility
Garbero et al. [100]	RCT	Standard care	113	Septic shock	No difference in overall mortality. Higher mortality in patients with severe renal failure		Terminated prematurely due to concerns of a possible harmful effect of CPFA

*AKI* acute kidney injury, *ARDS* acute respiratory distress syndrome, *CPB* cardiopulmonary bypass, *CPFA* coupled plasma filtration and adsorption, *CRP* C-reactive protein, *EAA* endotoxin activity assay, *ICU* intensive care unit, *IL* interleukin, *LOS* length of stay, *MAP* mean arterial pressure, *mHLA-DR* monocyte human leukocyte antigen, *PCT* procalcitonin, *RCT* randomised controlled trial, *RRT* renal replacement therapy, *SOFA* sequential organ failure assessment, *TNF* tumour necrosis factor, *TSA* trial sequential analysis

### Toramyxin

The Toramyxin cartridge (Toray Industries, Japan), a polymyxin B-immobilized (PMX-B) fiber column, is the most well-studied adsorber currently available. The Toraymyxin cartridge binds and neutralizes endotoxins, as polymyxin B coated fibres exert a high affinity for endotoxins [23]. With its mechanism of action focussed on endotoxins, its intended use is in patients with Gram-negative bacterial septic shock. This adsorber has been demonstrated to lower endotoxins levels in vitro as well as in animals in vivo, and was shown to improve clinical parameters such as blood pressure in a canine model of endotoxin-induced septic shock [23–25]. However, confirmation of these results in patients has proven to be troublesome. In 2009, the EUPHAS prospective randomized controlled trial (RCT) in 64 patients with severe sepsis or septic shock of abdominal origin was terminated prematurely due to early efficacy, showing that Toramyxin therapy reduced hazard ratios for in-hospital mortality up to 28-day in Cox regression models [26]. Importantly however, the 28-day mortality was not significantly different [27]. The mean arterial pressure (MAP) increased significantly after initiation of Toramyxin therapy, which was accompanied by a decrease in inotropic requirement and vasopressor dependency index, while this was not the case in the control group [26]. Finally, at 72 h after start of treatment, the Toramyxin group showed a greater reduction in SOFA score compared with the control group [26]. An international multicentre registry study (EUPHAS 2) reported similar beneficial effects [28], as did a retrospective propensity-matched cohort study from Japan including 1723 patients, in which a lower all-cause hospital mortality, but not ICU mortality was observed in the Toramyxin group (n = 522) compared to matched controls (n = 1201) [29]. In contrast however, larger prospective RCTs such as the ABDOMIX (n = 232) and EUPHRATES (n = 450) trials, found no improvements in haemodynamic parameters, organ dysfunction or survival in septic shock patients treated with Toramyxin compared to those receiving standard care [30, 31]. The EUPHRATES trial was a blinded, sham-controlled, and biomarker-guided trial (only patients with endotoxin activity assay (EAA) levels of 0.60 or higher were enrolled) where treatment with Toramyxin did not result in a more pronounced decrease in circulating endotoxin concentrations [31]. A RCT from Thailand in 59 patients showed that Toramyxin treatment (n = 29) improved monocyte human leukocyte antigen (mHLA-DR) expression, suggesting that this treatment may enhance immune competence of the host, but the limited number of included patients prevents any definitive conclusions [32]. Several meta-analyses on endotoxin removal devices, including Toramyxin, have been performed. One of these took disease severity into account, by stratifying (non-)RCTs according to the mortality observed in the conventional treatment group [33]. In this stratified meta-analysis, risk reduction was related to disease severity, as the most severely ill patients appeared to benefit most [33]. Another meta-analysis also reported a mortality benefit for endotoxin removal therapy, although results were highly variable between geographical regions [34]. Furthermore, a trial sequential analysis (TSA) performed on the same studies found no association between endotoxin removal and mortality [34]. In line with this TSA, other systematic reviews and meta-analyses concluded that there is still insufficient clinical outcome data to support routine use of Toramyxin [35–37]. The therapeutic efficacy may also be dependent on the concentration of circulating endotoxins. In a post-hoc analysis of the EUPHRATES trial, Toramyxin therapy appeared to exert more pronounced therapeutic efficacy in a subgroup of 194 patients with intermediate EAA levels (between 0.60 and 0.89) [38]. The authors hypothesised that an EAA ≥ 0.9 exceeds the binding capacity of the Toraymyxin adsorber and renders the two-session treatment regimen insufficient. Although several methodological aspects may have led to an overestimation of the treatment effect in this analysis [39], this post-hoc analysis illustrates the potential value of patient stratification: selecting those patients that may benefit the most from Toramyxin treatment. This approach is currently prospectively evaluated in the ongoing TIGRIS trial (NCT 03901807), in which 150 patients with endotoxemic shock and an EAA level of 0.60–0.89 will be randomized to either Toramyxin treatment on top of standard care or to standard care alone [40]. As of February 3rd 2025, 146 patients have been enrolled.

Drug removal from the circulation by the Toramyxin adsorber (or any other adsorption device) could have detrimental as well as beneficial effects. An example of the former is the removal of life-saving drugs such as antibiotics, necessitating dosing adjustments and therapeutic drug monitoring to ensure adequate plasma concentrations. Inadvertent removal of drugs could, however, also be beneficial in the context of intoxications or, e.g., when acute cardiac surgery is indicated in a patient that uses anticoagulants. Data on drug adsorption by the Toramyxin adsorber is still limited. An in vitro study showed that the PMX-B immobilized fibers used in the Toraymyxin adsorber highly adsorbed linezolid (22%) and, to a lesser degree, ciprofloxacin (6%) and piperacillin (4%) [41]. In an extracorporeal set-up, significant adsorption of linezolid and ciprofloxacin was also noted (55% and 7%, respectively) [42]. A small sub-study of a RCT [32] showed no significant removal of meropenem in eight critically ill patients treated with Toraymyxin and continuous venovenous haemofiltration (CVVH) [43].

### CytoSorb

The CytoSorb adsorber (CytoSorbents Inc., USA) contains polystyrene divinylbenzene copolymer beads coated with polyvinyl-pyrrolidone, which are designed to capture hydrophobic molecules with a molecular weight up to approximately 55 kDa, including several cytokines. In vitro, ex vivo and in vivo animal studies have shown that CytoSorb therapy lowers circulating concentrations of cytokines, but not of endotoxins [25, 44, 45]. Proof-of-principle was recently demonstrated in healthy volunteers undergoing experimental human endotoxemia, showing that CytoSorb therapy significantly lowers circulating cytokine concentrations [46]. This study also revealed saturation of the CytoSorb cartridge within several hours, as clearance rates decreased over time. In fact, clearance rate for some cytokines even became slightly negative over time, indicating desorption, a phenomenon that has also been observed for bilirubin, beta-lactam antibiotics and 3,4-methylenedioxymethamphetamine (MDMA) [47–50]. Again, results are difficult to reproduce in clinical patients. For instance, in 97 patients with sepsis, CytoSorb therapy was shown to capture interleukin (IL)-6, reflected by a gradient across the cartridge, but this did not result in lower plasma IL-6 concentrations compared to a control group receiving standard of care [51]. In addition, in a propensity score-matched analysis in 38 patients suffering from a cytokine storm caused by sepsis or severe trauma, CytoSorb therapy did not result in a significant reduction of plasma IL-6 concentrations compared with a matched control group [52]. Similarly, in 32 patients undergoing cardiopulmonary bypass (CPB) surgery, treatment with CytoSorb cartridges placed in the CPB circuit did not result in alterations of plasma cytokine concentrations compared to a control group [53]. Apart from the non-randomized nature of some of these patient studies and the fact that heterogeneity is a major issue limiting the power to demonstrate a change in circulating cytokine concentrations, they suffer from important additional shortcomings. First, concentrations of cytokines were quite low at baseline [51, 53], which is relevant as clearance by the cartridge is concentration-dependent. Second, plasma cytokine concentrations only show substantial increases several hours after CPB [54], rendering perioperative treatment with Cytosorb, i.e. in the period when cytokines are not yet elevated, not likely to be effective. Finally, the aforementioned saturation of the device combined with infrequent replacement of the cartridge and short duration of therapy in all studies performed to date is another factor that may explain the lack of therapeutic efficacy in these studies.

In the absence of adequately powered randomized controlled trials, it remains unclear whether CytoSorb therapy leads to haemodynamic stabilization and/or exerts therapeutic efficacy in improving organ dysfunction and survival. While multiple case series and a small randomized controlled pilot study including 20 patients with early onset of septic shock (< 24 h) showed a significant reduction in norepinephrine requirements at 48 h in the CytoSorb group compared to baseline [55], a recent meta-analysis including 2611 patients with sepsis revealed no differences in norepinephrine requirement between CytoSorb treatment and standard of care [56]. Furthermore, this meta-analysis showed no mortality benefit for CytoSorb therapy in various patient categories (i.e. sepsis, CBP surgery, severe illness, COVID-19, and cardiac arrest). Other meta-analyses that included only the few small RCTs that have been performed conclude that, despite a low to very low quality of evidence, CytoSorb therapy may rather be associated with increased mortality in inflamed critically ill patients [57, 58]. As the timing of initiation of CytoSorb therapy, as well as its duration and cartridge replacement intervals are unclear, conclusions on the efficacy as well as potential harm of CytoSorb therapy should be interpreted with great caution [59].

Regarding drug removal, an in vitro study [60] and an in vivo study in pigs [49] concluded that CytoSorb therapy enhances the clearance of several antibiotic and antifungal drugs, especially during the first hours of treatment. Nevertheless, this still needs confirmation in patients, although a small observational study concluded that an additional dosage of vancomycin was required to ensure therapeutic plasma concentrations in seven patients with sepsis or septic shock [61]. On the positive side, a nonrandomized study revealed that CytoSorb therapy during acute cardiac surgery (cartridge placed in the CPB circuit) in patients treated with ticagrelor or rivaroxaban significantly reduced the risk of postoperative bleeding complications compared to a group of patients receiving standard of care [62]. Possibly related to these beneficial effects, also a shorter duration of surgery, as well as ICU- and in-hospital length of stay (LOS) was observed in the CytoSorb group [62].

### Jafron (HA330 and HA380)

The HA330 and HA380 cartridges (Jafron Biomedical Co. Ltd., China) contain neutro-macroporous resin-adsorbing beads made of a styrene–divinylbenzene copolymer intended for use in hyperinflamed critically ill patients [63]. The HA380 is similar to the HA330 cartridge; the only distinction is that it contains more beads. Both cartridges are designed to remove several inflammatory mediators, including cytokines, based on molecular weight (10 to 60 kDa). An in vitro study showed that the HA380 cartridge is capable of removing several cytokines, but removal was less pronounced and slower compared with the CytoSorb cartridge [64]. The recommended treatment duration is 2–2.5 h after which the cartridge is saturated [65]. In 2010, a study randomized 44 patients with sepsis to HA330 treatment (once per day for 2 h on 3 consecutive days) on top of standard care or to standard care alone [66]. Circulating concentrations of IL-6 and IL-8 in the HA330 group were significantly attenuated compared to the control group after 3 days of treatment. Furthermore, several haemodynamic parameters (e.g. MAP, cardiac index) on days 3 and 7 were significantly better in the HA330 group compared to the control group. Finally, a lower SOFA score as well as a shorter ICU-LOS and lower ICU mortality were observed in the HA330 group compared with the control group. Another randomized study in 46 patients with acute respiratory distress syndrome as a consequence of extrapulmonary sepsis revealed similar beneficial effects of HA330 treatment (also performed once per day for 2 h on 3 consecutive days) on dopamine and norepinephrine requirements, as well as on ICU-LOS, ICU mortality, and 28-day mortality [67]. In addition, the decrease in plasma concentrations of IL-1 and tumour necrosis factor (TNF) was significantly more pronounced in the HA330 group on day 3 compared to the control group. Nevertheless, the sample size of these trials is small and confirmation is required.

To the best of our knowledge, evidence is scarce for the use of the HA380 cartridge in patients with sepsis. Most evidence regarding its efficacy has been obtained in patients undergoing CPB surgery [68]. Recently, a RCT in 60 patients undergoing CPB surgery for valve replacement showed that use of the HA380 cartridge on top of standard care (n = 30) compared to standard care alone resulted in lower plasma IL-6 concentrations and vasoactive inotropic score in the first 24 h, and a reduction in ICU LOS and duration of mechanical ventilation [69]. A retrospective study in 117 patients with acute type A aortic dissection also showed that treatment with the HA380 cartridge (n = 60) resulted in lower plasma IL-6 concentrations in the acute postoperative period compared to standard care. Furthermore, in the HA380 group the incidence of acute kidney injury (AKI) and acute respiratory distress syndrome (ARDS) was lower compared to the control group (25.4% vs. 44.6%, p = 0.03 and 18.3% vs. 35.1%, p = 0.04, respectively) [70]. Other postoperative complications such as bleeding did not differ between the groups, and this was also the case for mortality and ICU or hospital LOS. Of note, the putative beneficial effects on AKI and ARDS should be interpreted with caution, as the fragility index [71] shows that if one or even no additional patients in the HA380 group had developed AKI or ARDS respectively, statistical significance would be lost.

Although another Jafron cartridge, the HA230, is primarily indicated for acute intoxications, the HA330 cartridge may also be effective in colchicine intoxication and paraquat poisoning [72, 73]. Furthermore, in vitro and in vivo animal studies showed that the HA380 cartridge removes vancomycin [74, 75], gentamicin [75], meropenem [76], and piperacillin [76], indicating that therapeutic drug monitoring is of importance in patients with sepsis treated with the HA380 cartridge.

### oXiris

The oXiris haemofilter (Baxter, France) contains a copolymer combining acrylonitrile and sodium methallylsulfonate molecules with a coating of polyethyleneimine [77]. The membrane is also grafted with heparin for antithrombogenic properties. In contrast to the abovementioned adsorption devices, oXiris is designed to be used for conventional renal replacement therapy (RRT), as well as to remove endotoxins and cytokines. The polyethyleneimine coating enables the adsorption of negatively charged molecules such as endotoxins, whereas the sulfonate groups enable cytokine adsorption [77]. In vitro*,* oXiris was demonstrated to remove endotoxins and inflammatory mediators to a similar extent as the Toraymyxin and CytoSorb cartridges, respectively [25]. oXiris treatment has been associated with favourable outcomes in several case series [77]. A retrospective cohort study in 31 patients with septic shock requiring RRT observed a significant improvement in haemodynamics and lactate in the first 48 h after start of treatment with the oXiris haemofilter, especially in a subgroup of patients with abdominal septic shock or those affected by gram-negative bacteria [78]. In a randomized crossover trial in 16 patients with sepsis shock and AKI who were treated for 24 h with either the oXiris haemofilter or a standard filter in addition to continuous venovenous haemodiafiltration (CVVHDF), oXiris treatment led to a significantly more pronounced decrease in circulating endotoxin concentrations compared to treatment with the standard filter [79]. Furthermore, although plasma concentrations of TNF, IL-6, IL-8 and interferon-γ decreased over time in both groups, this decrease was more pronounced in the oXiris group. Blood lactate levels and norepinephrine requirements were also significantly lower in the oXiris group compared to the standard filter group. Nevertheless, many of these differences may be ascribed to baseline differences between the groups and regression to the mean, as endotoxin levels in the oXiris group tended to be higher than those in the standard filter group (p = 0.06), as were cytokine concentrations and norepinephrine infusion rates. A recent study in 30 patients with severe septic shock and an EAA ≥ 0.6 showed no significant difference in endotoxin adsorption after 48 and 72 h using the oXiris haemofilter (or Toraymyxin cartridge) compared to standard of care [80]. A meta-analysis in 695 patients with sepsis on RRT (including 10 observational studies with a control group and 4 RCTs) concluded that oXiris treatment is associated with a lower 28-day mortality and shorter ICU-LOS, although ICU mortality, hospital and 90-day mortality, and hospital-LOS were similar compared to the control group [81]. In addition, this meta-analysis revealed that oXiris treatment was associated with a more pronounced attenuation in circulating IL-6 and lactate concentrations, as well as with a reduction in SOFA score and norepinephrine requirements, and these parameters were comparable between the active and control groups at baseline. However, as the authors partly acknowledge, the quality of the evidence was low to very low since most included studies were observational in nature with no clear description of the control group, not all studies were peer-reviewed, and the few RCTs all had a small sample size. Also, there are methodological issues with the meta-analysis itself, thus these results should be interpreted with caution [82]. A more recent meta-analysis including 481 patients with sepsis and COVID-19 (2 RCTs and 8 non-randomized prospective and retrospective studies) also revealed that treatment with the oXiris haemofilter is associated with a reduction in overall mortality compared to the control group [83]. However, the certainty of evidence was once again judged to be very low. The most recent trial on oXiris was performed in patients undergoing cardiac surgery, who were randomized to oXiris (placed in the CPB circuit, (n = 169) or standard care (n = 174) [84]. This study demonstrated a significantly lower incidence of AKI in the oXiris group in the first 7 days after randomization (28% vs. 40% in the standard care group, p = 0.03). There were no significant differences in survival and ICU or hospital LOS. Furthermore, the efficacy of oXiris therapy on renal function is still up for debate, as (peak) serum creatinine concentrations and oliguria, both used to specify the KDIGO criteria, were similar between the groups, as was RRT requirement. Also, the fragility index [71] indicates that if as few as two additional patients in the intervention group would have developed AKI, statistical significance would be lost. In any case, it is unclear whether the putative renal protective effect of the oXiris haemofilter is mediated by removal of endotoxins and/or cytokines, or by its RRT properties.

To our knowledge, no studies have evaluated drug removal by the oXiris haemofilter. One can speculate that removal of antibiotics could be an issue with the oXiris haemofilter through diafiltration or adsorption, as the oXiris haemofilter shares structural similarities with the AN69ST haemofilter [85]. An in vitro study showed significantly more adsorption of vancomycin, gentamycin and tigecycline during CVVH with the AN69ST haemofilter compared to a control filter [86].

### Other devices

Apart from the above described therapies, several more recently developed adsorption devices are available and under investigation. For instance, the Alteco LPS adsorber (Alteco Medical AB, Sweden) is a cartridge containing porous polyethylene plates with covalently bound cationic peptides that capture negatively charged LPS (lipopolysaccharide) [87]. An observational study in 24 patients with septic shock showed that treatment with the this adsorber was associated with a significantly shorter duration of norepinephrine infusion compared to a matched historical control group [87]. However, use of the adsorber was also associated with a significant loss of thrombocytes, requiring transfusion in two patients. RCTs on the Alteco LPS adsorber in patients with sepsis have been terminated prematurely due to a lack of efficacy (observed in an interim analyses after 15 patients completed the trial) [88] or a low enrolment rate [89].

In contrast to the currently available adsorption devices that remove endotoxins and/or cytokines, a novel device called the Seraph-100 Microbind Affinity Blood Filter (ExThera Medical, USA) is designed to remove various pathogens and cytokines from the bloodstream [90]. The Seraph-100 filter contains polyethylene beads coated with negatively charged heparan sulphate, which can bind positively charged pathogens, including several Gram-positive and Gram-negative bacteria, viruses (e.g. SARS-CoV-2), fungi and cytokines due to electrostatic interactions [90, 91]. In vitro experiments have shown that the Seraph-100 filter also eliminates antibiotic, antiviral and antifungal agents*.* For instance, it reduced tobramycin and gentamycin concentrations by 62% and 59%, respectively [92]. The first-in-human non-randomised study included 15 chronic haemodialysis patients on RRT with blood culture-positive bloodstream infections who all were treated with Seraph-100 [93]. In these patients, 4 h of Seraph-100 treatment was not associated with the occurrence of adverse events. Furthermore, a reduction in bacterial load was observed in four patients after 4 h of treatment, which is however difficult to interpret in the absence of a control group and the small sample size [93]. During the COVID-19 pandemic, the U.S. Food and Drug Administration granted emergency use authorization for Seraph-100 in critically ill COVID-19 patients with confirmed or imminent respiratory failure. Several case series and an interim analysis of a registry revealed that Seraph-100 treatment appears safe and well-tolerated in patients with COVID-19 and might improve clinical parameters [94–97]. A recent retrospective multicentre study (n = 106) confirmed the safety and efficacy of Seraph-100 therapy in 53 patients with severe COVID-19 compared to a contemporary control group (n = 53) [91]. Furthermore, it was associated with more vasopressor-free days (25 vs. 15) and lower mortality (32% vs. 64%). However, the increase in vasopressor-free days did not reach statistical significance in a multivariable model and the reduction in mortality was not evidenced when patients treated with Seraph-100 were compared to a control group of an external cohort [91]. Again, confirmation in an adequately powered RCT is warranted.

### Coupled plasma filtration and adsorption (CPFA)

CPFA consists of two stages, a plasma separation and a haemofiltration stage [98]. Blood flows initially through a plasma filter after which the separated plasma is passed through an adsorption device to remove inflammatory mediators and/or toxins. Subsequently, the plasma is reinfused before the haemofilter for the haemofiltration stage which removes small water-soluble molecules. Two large multicentre RCTs in patients with septic shock (COMPACT and COMPACT-2) were performed [99, 100]. In the COMPACT trial, patients with septic shock were randomised to CPFA treatment on top of standard care or to standard care alone [99]. After including 192 patients, the trial was prematurely terminated based on futility, concerns regarding the low recruitment rate and excess protocol violations in the CPFA arm. In total, 184 patients were analysed (CPFA group: n = 91; control: n = 93), of which 48% of patients who received CPFA treatment were undertreated, mainly due to clotting of the circuit. No significant difference in hospital mortality was found, as well as the occurrence of new organ failures and ICU free days within the first 30 days. A subgroup analysis revealed a potential mortality benefit in patients in whom a larger plasma volume was treated. Therefore, in the COMPACT-2 trial, patients with septic shock were randomised to high dose CPFA (> 0.2 L/kg of treated plasma per day) on top of standard care versus standard care alone [100]. At the first interim analysis, aimed at assessing the feasibility of high dose CPFA, it became apparent that several patients deceased very quickly after initiation of CPFA. This led to another interim analysis to assess 3-day mortality, which confirmed the previous concerns of a possible harmful effect of CPFA. Hence, the study was prematurely terminated after including only 113 patients. Finally, a meta-analysis concluded that CPFA treatment does not decrease all-cause mortality in patients with septic shock [101]. Taken together, it is currently not recommended to use CPFA for the treatment of septic shock.

## Potential downsides of current adsorption-based therapies

Most therapies that are currently available employ adsorption based on size, charge, and/or hydro/lipophilicity. Therefore, they are non-specific to a certain degree, resulting in the removal of a relatively broad spectrum of molecules. This may potentially influence the therapeutic efficacy of the treatment, as inflammatory mediators which do not play a detrimental role, or are even beneficial, at least in some conditions or disease stages, will also be removed. Furthermore, as briefly touched on before, these therapies may also remove other essential therapeutic compounds, such as antibiotics. Therefore, selective removal of inflammatory mediators could prove to be superior.

## Selective haemoadsorption: an option for the future?

Haemoadsorption based on magnetism has been proposed as a selective technique to remove inflammatory mediators from the blood. This method is based on high-gradient magnetic separation in conjunction with antibodies against a target of interest which are exposed to blood in an extracorporeal circuit, after which the exposed blood passes through a strong magnet which retains the particles with the bound target [102, 103]. A similar technique has been used for many decades in laboratories where functionalised magnetic particles bind to specific cell types and remove those from a heterogenic mixture [104].

**Fig. 2:**
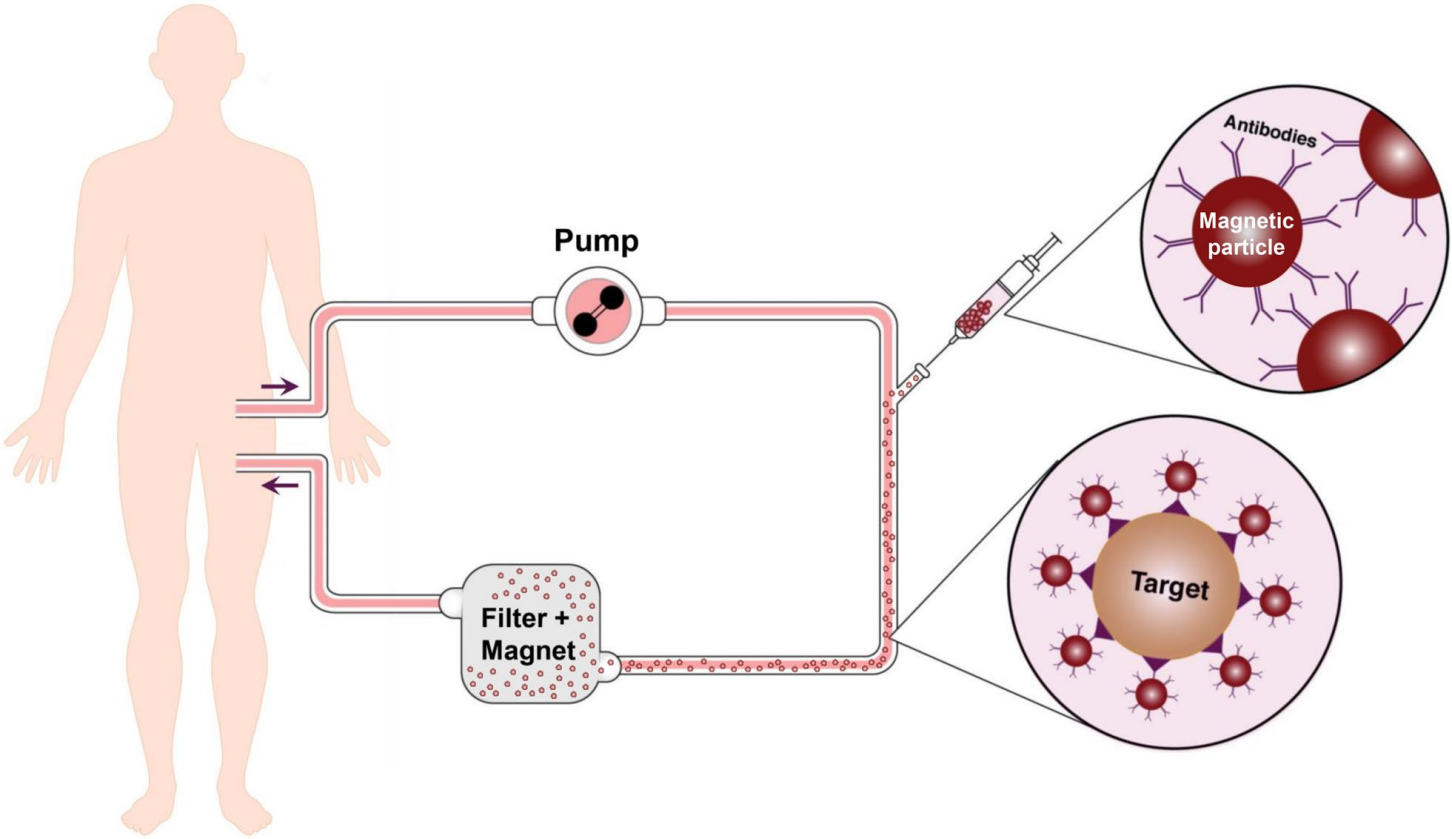
Schematic overview of the Sieve system

The IL-6-Sieve haemofiltration system (MediSieve Limited, UK), employs such an approach, using antibodies directed against the cytokine IL-6 coupled to magnetic beads. It comprises four component devices: Filter, Magnet, Bead Adapter and Anti-IL-6 Beads. The Anti-IL-6 Beads are infused into an extracorporeal circuit via a Bead Adapter, after which they are captured in a Filter placed inside a Magnet, before the blood returns to the bloodstream of the patient (see Fig. 2). This extracorporeal treatment will lead to the selective removal of IL-6 from the bloodstream. IL-6 is a crucial cytokine in the pathogenesis of hyperinflamed critically ill patients with sepsis, COVID-19, and the cytokine release syndrome (CRS) that a proportion of patients develop after receiving CAR-T cell therapy [105, 106]. For the latter two conditions, tocilizumab, a recombinant humanised IgG1 monoclonal antibody against the soluble and membrane-bound IL-6 receptor, is currently part of standard care. However, due to its long half-life, tocilizumab supresses the immune response for several weeks, potentially rendering the patient susceptible to secondary infections [107]. Therefore, the IL-6-Sieve could be an interesting treatment option for these patients, as it only removes IL-6 for the duration of treatment and does not lead to long-term neutralization of IL-6 signalling. Recently, the first-in-human study in six healthy volunteers revealed that use of the IL-6-Sieve’s Filter and Magnet component devices placed in an extracorporeal circuit (i.e. without Anti-IL-6 Beads and the Bead adapter) is safe and well-tolerated [108]. In the next paragraph, we report on the cytokine capturing potential of the IL-6-Sieve in a benchtop experiment.

## In vitro data on selective cytokine capture by the IL-6-Sieve

**Fig. 3:**
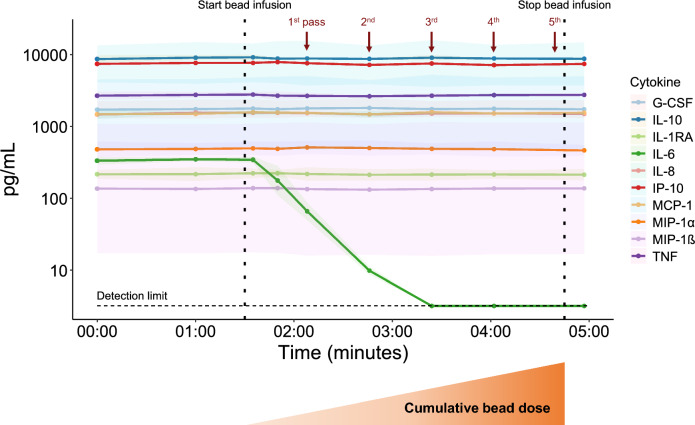
Cytokine removal by the IL-6-Sieve in an in vitro experiment. Data are presented as median (coloured lines) with interquartile range (shaded areas) of the four plasma samples. Dotted vertical lines at 1:30 min and 4:45 min mark the start and cessation of Anti-IL-6 Bead infusion, respectively. The dashed horizontal line at 3.2 pg/mL indicates the lower limit of detection for all cytokines

Four human plasma samples (Research Donors, UK) were spiked with known concentrations of several pro- and anti-inflammatory cytokines (see Table 2). Subsequently, each plasma sample was run through a circuit at a flow rate of 120 mL/min for 5 min, resulting in a total of 5.2 passes. Anti-IL-6 Beads were infused via the Bead Adapter into the circuit (0.6 mL/min) before the plasma entered the Filter placed inside the Magnet. Bead infusion was initiated 1.5 min after start of circulation. Samples were obtained at several timepoints, vortexed, and centrifuged (3500*g* for 10 min), after which plasma was stored at − 20 °C until batchwise analysis of cytokine concentrations at the Radboud university medical center (the Netherlands). Concentrations of TNF, IL-1RA, IL-6, IL-8, IL-10, MIP-1α, MIP-1ß, MCP-1, G-CSF, and IP-10 were determined using a simultaneous Luminex assay (Milliplex, Millipore, Billerica, USA) as per the manufacturer’s instructions (lower limit of detection was 3.2 pg/mL for all cytokines). As shown in Fig. 3, after initiation of Anti-IL-6 Bead infusion, plasma concentrations of IL-6, but not of the other cytokines, rapidly declined. The data illustrates that the IL-6-Sieve indeed selectively removes IL-6 without affecting other inflammatory mediators.

**Table 2:** Overview of the cytokines used to spike the human plasma samples

Cytokine	Concentration (ng/mL)	Supplier
TNF	1.5	Thermo Fisher Scientific, USA
IL-1 receptor antagonist (IL-1RA)	1.5	PeproTech, Thermo Fisher Scientific, USA
IL-6	1	Sigma-Aldrich, USA
IL-8	1.5	PeproTech, Thermo Fisher Scientific, USA
IL-10	3	PeproTech, Thermo Fisher Scientific, USA
Macrophage inflammatory protein (MIP)-1α	1.5	PeproTech, Thermo Fisher Scientific, USA
MIP-1ß	7	R&D Systems, USA
Monocyte chemoattractant protein (MCP)-1	7	PeproTech, Thermo Fisher Scientific, USA
Granulocyte colony-stimulating factor (G-CSF)	3	PeproTech, Thermo Fisher Scientific, USA
Interferon-γ-induced protein (IP)-10	3	PeproTech, Thermo Fisher Scientific, USA

*IL* interleukin, *TNF* tumour necrosis factor

## Applicability of the system beyond IL-6 removal

The principle on which the IL-6-Sieve is based is versatile, as antibodies against potentially any target could be developed and coupled to the magnetic beads. In the case of sepsis, beads targeted against endotoxins or other important cytokines could also be employed in this system to remove excessive concentrations. Generally speaking, several diseases currently treated with plasmapheresis could also be managed with a much more specific ‘Sieve’ system [109, 110]. Another potential application for a Sieve system lies in adeno-associated virus (AAV) gene therapy, in which AAV is used as a vector for gene delivery. AAV transduction and therefore efficacy of this type of gene therapy is diminished by the presence of neutralizing antibodies (Nab) [111]. Individuals who are infected with wildtype AAV often develop these Nabs, so removing them by a Sieve could improve the efficacy of AAV gene therapies.

## Other future perspectives

As discussed in this review, numerous studies have evaluated the effects of haemoadsorption in critically ill patients with sepsis or in those undergoing cardiac surgery. However, most of these studies are not adequately powered and/or have relevant design-flaws related to the selection of patients and timing and dosing of these treatments, preventing clear assessment of their efficacy of this type of treatment. Therefore, adequately powered and well-designed randomised-controlled trials are urgently needed in the future. These studies should pay careful attention to the selection of patients, as well as to the timing, duration and dosing (timely replacement of the adsorbers) of therapy. Ideally, a precision medicine approach using a combination of prognostic and predictive enrichment should be employed to tailor this type of therapy to those who are most likely to benefit [112–114]. Prognostic enrichment identifies subgroups of patients who are at high risk of a clinically relevant outcome (e.g. mortality, incidence of AKI), whereas predictive enrichment identifies subgroups of patients who are likely to respond to treatment based on their underlying pathophysiology (e.g. the use of immunosuppressants in patient with hyperinflammation). Predictive enrichment is challenging in sepsis, due to the dynamic and complex nature of the dysregulated immune response [115], and highly depends on accurate biomarkers to select the right patients for therapy. The aforementioned TIGRIS trial is an example of a study using predictive enrichment in the field of haemoadsorption. Next to increasing the body of evidence concerning the relatively non-specific therapies currently available, future studies are required to investigate the safety and efficacy of the IL-6 Sieve in patients.

## Conclusion

Haemoadsorption in patients with sepsis or septic shock is potentially beneficial. However, based on the current literature its routine use is not (yet) recommended. High quality research into existing and novel treatment modalities is urgently warranted to move this field forward.

## Data Availability

The datasets used and/or analysed during the current study are available from the corresponding author on reasonable request.

## Abbreviations

AAV: Adeno-associated virus
AKI: Acute kidney injury
ARDS: Acute respiratory distress syndrome
CRS: Cytokine release syndrome
CVVH: Continuous venovenous haemofiltration
CVVHDF: Continuous venovenous haemodiafiltration
CPFA: Coupled plasma filtration and adsorption
EAA: Endotoxin activity assay
EBP: Extracorporeal blood purification
G-CSF: Granulocyte colony-stimulating factor
HVHF: High-volume haemofiltration
ICU: Intensive care unit
IL: Interleukin
IP: Interferon-γ induced protein
IL-1RA: Interleukin-1 receptor antagonist
LOS: Length of stay
LPS: Lipopolysaccharide
MAP: Mean arterial pressure
MCP: Monocyte chemoattractant protein
MDMA: 3,4-Methylenedioxymethamphetamine
mHLA-DR: Monocyte human leukocyte antigen
MIP: Macrophage inflammatory protein
Nab: Neutralizing antibody
PMX-B: Polymyxin B
RCT: Randomized controlled trial
RRT: Renal replacement therapy
TNF: Tumour necrosis factor
TSA: Trial sequential analysis
